# An Analysis of the Learning Health System in Its First Decade in Practice: Scoping Review

**DOI:** 10.2196/17026

**Published:** 2020-03-19

**Authors:** Jodyn E Platt, Minakshi Raj, Matthias Wienroth

**Affiliations:** 1 Department of Learning Health Sciences University of Michigan Medical School Ann Arbor, MI United States; 2 Department of Health Management and Policy University of Michigan School of Public Health Ann Arbor, MI United States; 3 School of Geography, Politics & Sociology Newcastle University Newcastle upon Tyne United Kingdom

**Keywords:** learning health system, review, knowledge management, bioethics, health information exchange

## Abstract

**Background:**

In the past decade, Lynn Etheredge presented a vision for the Learning Health System (LHS) as an opportunity for increasing the value of health care via rapid learning from data and immediate translation to practice and policy. An LHS is defined in the literature as a system that seeks to continuously generate and apply evidence, innovation, quality, and value in health care.

**Objective:**

This review aimed to examine themes in the literature and rhetoric on the LHS in the past decade to understand efforts to realize the LHS in practice and to identify gaps and opportunities to continue to take the LHS forward.

**Methods:**

We conducted a thematic analysis in 2018 to analyze progress and opportunities over time as compared with the initial *Knowledge Gaps and Uncertainties* proposed in 2007.

**Results:**

We found that the literature on the LHS has increased over the past decade, with most articles focused on theory and implementation; articles have been increasingly concerned with policy.

**Conclusions:**

There is a need for attention to understanding the ethical and social implications of the LHS and for exploring opportunities to ensure that these implications are salient in implementation, practice, and policy efforts.

## Introduction

### Background

In 2007, Lynn Etheredge [1] envisioned the Learning Health System (LHS) in which he described a system aimed at increasing the value of health care without *draconian* cost cutting. He encouraged facilitating what he called *rapid learning* from new evidence for practice and policy. The publication of this first article to explicitly use the language of the LHS and urge the further consideration of such a system coincided with the adoption of electronic health records (EHRs) in clinical settings aimed at integrating clinical, financial, and administrative data [1]. Etheredge [1] described several opportunities for answering key questions about population health and health care delivery. Addressing these questions would demand a change in institutional (eg, Medicaid) and organizational (eg, in hospitals) leadership and in funding structures to advance the use of health information to improve health [1]. Etheredge’s proposals envisioned competitive markets led by health plans and providers who use EHRs; payment linked to evidence-based protocols; and Medicaid and the State Children’s Health Insurance Program as national leaders in EHR adoption and in the use of EHR research databases. National computer-searchable clinical trial databases and national assessments of new technologies would support these efforts. Here, we considered Etheredge’s article [1] as an initial conceptualization of gaps and opportunities for future examination and development of the LHS. Many subsequent articles on the LHS topic have cited the guiding principles presented in Etheredge’s article [1,2].

Over a decade has passed since this early vision. Health care provision generates significant amounts of patient and experiential data, and health records, laboratory results, population health surveillance, and patient-generated data, that can be agglomerated and analyzed within health systems. These activities are the result of (1) an increase in health data availability within growing information technology systems via the widening use of patients’ EHRs; (2) efforts to increase the volume of clinical research with patients undertaken at health care facilities; and (3) considerable research and data generation by government-funded and commercial enterprises [3-8]. Bringing together diverse actors in the health and life sciences context, LHSs aim to gather and analyze differently sourced data to create useful knowledge that is disseminated to all stakeholders, put into practice, and then evaluated [9].

The LHS framework marks a departure from data practices that are governed by the intended use for data, be it research, quality improvement (QI), clinical care, or public health. The aim of LHS is to enable continuously and rapidly operating virtuous cycles of study, feedback, and practice change, regardless of the original intention for data collection; its vision has come to shape the goals of initiatives in the United States, the United Kingdom, and globally [1,2]. Examples include the American Society of Clinical Oncology’s CancerLinQ initiative, which offers an emerging large-scale database for an oncology learning community to improve the quality of cancer care [10], the CommonWell Health Alliance, which enables querying of treatment data for over 17 million unique individuals [11], and the National Institutes of Health *All of Us* research initiative, which seeks to connect genomic, health, and social media data from over 1 million individuals [12]. Similar avenues toward large-scale agglomeration, analysis, and sharing of patient health information exist in the United Kingdom, such as the National Health Service–supported data platform, Lambeth DataNet, which provides general physicians and academic researchers with access to data from approximately 350,000 patients [13] and the Connected Health Cities initiative [14], which is building data stores to support direct care across the North of England region.

### Objectives

**Figure 1:**
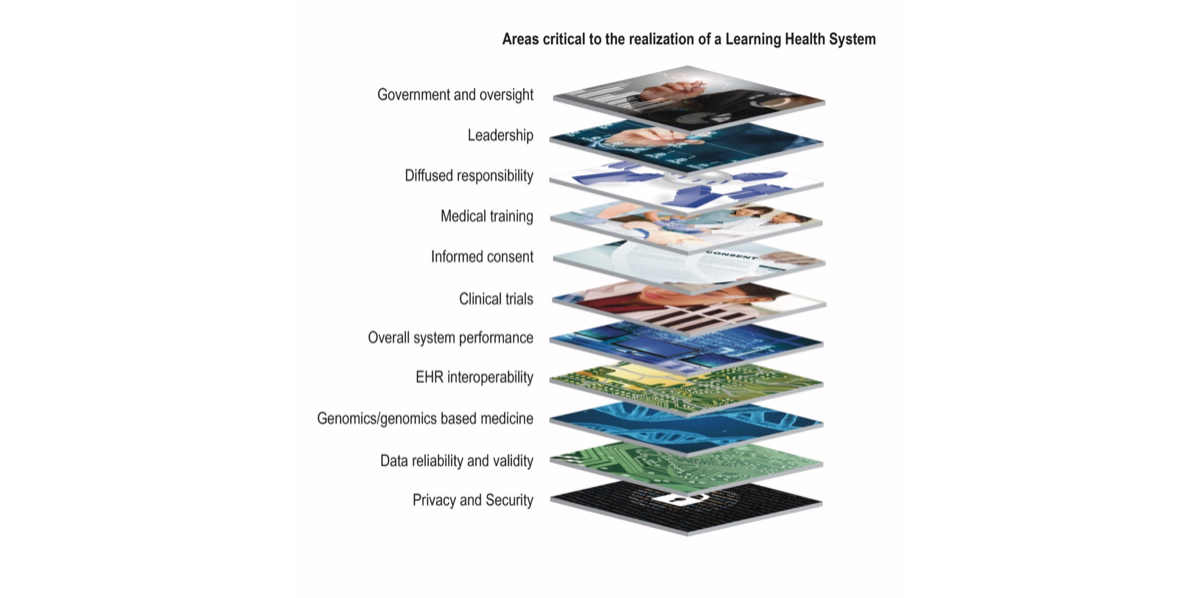
Areas critical to the realization of a learning health system.

In this review, we aimed to examine the literature and rhetoric on the LHS, identify trends and themes in published efforts undertaken to moving this idea from concept to reality, and assess gaps in the literature that suggest areas for future research and policy considerations that are necessary for moving the LHS forward (Figure 1).

## Methods

### Search Strategy

We conducted a scoping review using search terms “learning health system(s),” “learning health care system(s),” and “learning healthcare system(s)” on PubMed, Web of Science, and Scopus databases to identify peer-reviewed publications. The search was limited to peer-reviewed articles published in the English language between January 2007 and December 2017.

We identified and included articles published in a variety of clinical contexts, with most articles focusing on the United States and written by US-based authors and some focused on the United Kingdom, India, Sweden, Kenya, and China. Articles describing or examining the LHS from clinical, technical, and ethical perspectives were included. Our initial search yielded 542 articles for review, including USA Institute of Medicine (now USA National Academy of Medicine) proceedings and chapters that informed our search but were not included in the final full analyses.

### Data Extraction and Article Selection

Following a title and abstract review, we excluded 222 articles. Articles were excluded if they were duplicates, conference proceedings or posters, or not peer reviewed. Results were compared among members of the study team before exclusion. We created a Microsoft Excel spreadsheet charting information from the 320 remaining articles, including (1) article title; (2) year; (3) country; (4) category; (5) concern or focus; (6) field; and (7) number of papers citing the article of interest (Table 1).

**Figure 2:**
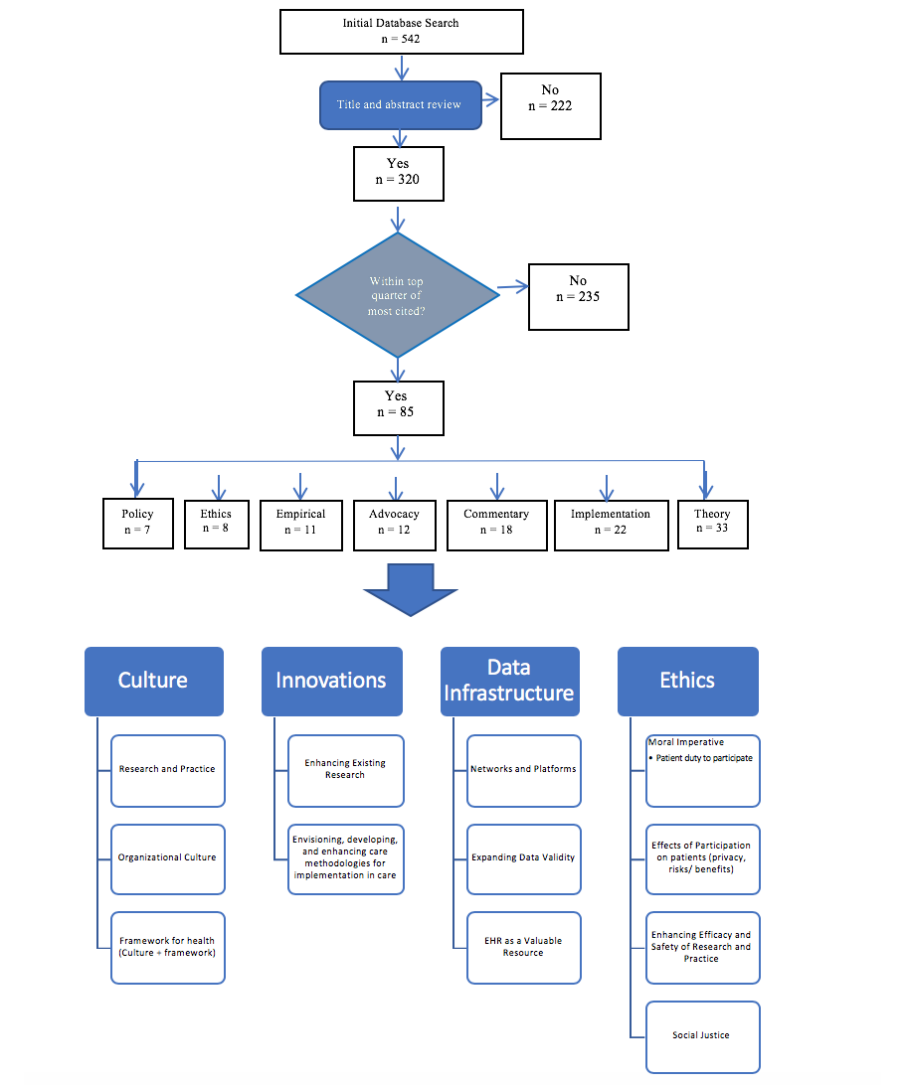
Summary of search strategy and themes.

Next, we sorted articles by year, identified the top quarter of most cited papers within each year. We used citation data provided by Web of Science and Scopus to determine the number of citations for each article, sorted all included articles in the sample to identify the top quarter, and then compiled a list of these articles. We chose to focus on the most cited papers as representatives of those with the most salient themes to the discourse and scholarship on the LHS. This sorting resulted in 85 articles for our final review, which represents just over 15.7% (85/542) of the total number of articles identified in our initial search (see Figure 2). The 85 articles were read in full to conduct a more thorough discourse (thematic mapping) analysis that could be compared with the vision laid out in Etheredge’s [1] manuscript in 2007, which is described in the section, *Analytical Strategy*.

**Table 1:** Summary of abstracted information.

Information documented	Description
Article title	Title of publication
Year	Year of publication
Country	Global context of publication
Category	Preset categories for thematic understanding, including the following: Policy (eg, relating to health reform) Advocacy (eg, encouraging implementation into organizations) Theory (ie, generating or enhancing LHS^a^ frameworks) Empirical (eg, studies testing infrastructures or hypotheses) Implementation (ie, evaluating implementation into clinical contexts) Ethics (ie, introducing or describing ethical perspectives or limitations of the LHS) General commentary
Concern and/or Focus	Information on the primary focus or concern of the article, such as the following: Quality improvement Personalized health care Evidence-based medicine Electronic health record Ethical oversight
Field	Information on perspective through which article discusses the LHS, such as the following: Clinical context (eg, oncology and surgery) Professional context (eg, nursing) Discourse context (eg, medical informatics, research, and ethics) Health care system (ie, articles discussing the LHS across a larger scope)
Number of citations	Number of times each article has been cited according to Web of Science or Scopus

^a^LHS: Learning Health System.

### Analytical Strategy

The analysis presented in this paper draws on both quantitative and qualitative approaches. First, we identified historical trends in the LHS discourse by quantitatively assessing the number of articles published on the LHS over time. We then evaluated article categories over time and assessed the variety of clinical domains that have integrated the LHS discourse for the 85 articles selected for review.

Next, we performed a thematic mapping analysis to qualitatively examine the variety of ways in which articles define the LHS. This is a method for extracting, analyzing, and reporting themes in data, such as from interviews or texts [15]. Themes describe textual data that are grouped around a main issue [16] and are recurrent and systematic [17]. Moreover, themes emerge from the data and can be refined into levels or subthemes that reflect on the theme [18]. In this review, we initially identified broad themes that emerged from the articles. These themes were then refined into subthemes. Finally, we compared the qualitative findings from our literature review with the vision developed by Etheredge [1] in 2007 to assess the extent to which these initiatives have been undertaken and discussed according to the literature over the past decade and to identify areas for continued effort. One member of the study team coded the 85 articles using the MAXQDA software and shared codes and code relation matrices with the other members to ensure agreement. The study team held meetings every 2 weeks to discuss emerging themes and subthemes, thus arriving at the final list iteratively and collaboratively.

## Results

### Defining the Learning Health System

Articles in our final sample most commonly draw upon the Institute of Medicine’s (now the National Academy for Medicine) ambitious and encompassing definition describing LHSs as those that: *generate and apply the best evidence for the collaborative health care* *choices of each patient and provider;…drive the process of discovery as a natural outgrowth of patient care; and ensure innovation, quality, safety, and value in health care* [19].

These articles describe the LHS as an avenue for delivering more targeted, safe, and effective health care by using information from the experiences and treatment of patients to inform decision making and subsequent care in real time [20,21]. A central ambition, as well as a vehicle for the aspirations of the LHS, is the notion of *learning*. Importantly, learning itself is considered the transfer of knowledge through formal curricula (eg, during medical training) and a transfer of culture, attitudes, and beliefs in ways that can be implemented in research as well as practice when combined with QI methods [21,22]. For example, Faden et al [23] describe learning as a process including research, information gleaned from QI efforts, and comparative effectiveness research, culminating in improved practice [24]. In this way, QI is described as a critical stepping stone to learning, which depends on research and also has the potential to bridge the traditional gap between research and practice to inform the goals of an LHS [24].

Some articles refer to the LHS more specifically, as a *rapid learning health system* to emphasize the celerity—eg, *real-time* delivery—with which evidence-based medicine can be used to determine health care decisions [1,25]. A system, in this context, is characterized as requiring a coherent, flexible organizational structure, with data maintained in a repository until needed for a particular purpose [26]. In addition, mechanisms are in place to ensure that data are usable by all entities within the system and that they are transferred appropriately, safely, and ethically. Data originate from clinical practice, research, participation, and inquiry provided by organizational leaders, physicians, researchers, patients, and research participants [26]. Although some publications in our sample offer a discussion of the LHS as a large-scale system spanning borders, others implement, evaluate, or present challenges on the system at a local or regional scale.

### Quantitative Trends in the Learning Health System Discourse

**Figure 3:**
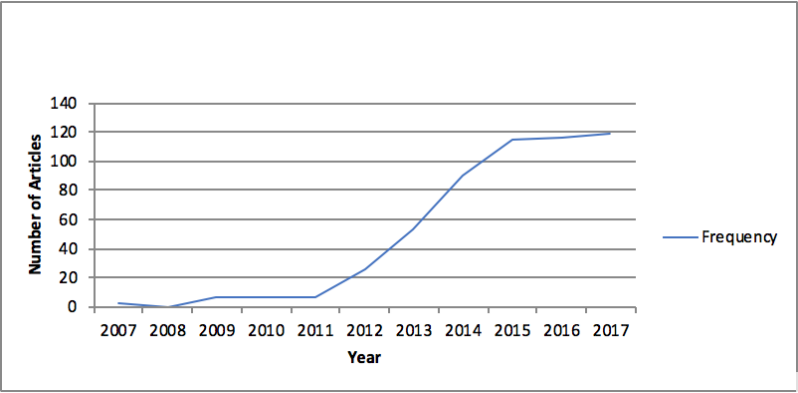
Frequency of articles published by year (n=542).

Our review indicated historical trends in the LHS discourse, with a particular surge in articles published between 2013 and 2017. This trend, we noticed in the broader literature search, is consistent with the trends in articles we selected for review (Figure 3).

Our review resulted in the selection of 85 articles published between 2007 and 2017. Most of the 85 articles included in our review were published in 2014, and the articles cited most were published in 2010 (132 citations); 2007 (116 citations); and 2013 (102 citations).

### Trends in Categories of Articles

**Figure 4:**
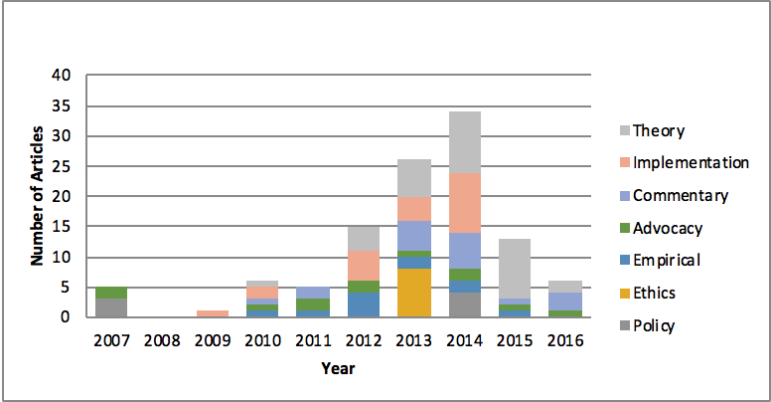
Trends in article categories among frequently cited articles based on review of frequently cited articles (n=85).

Following the categorization of each paper according to its primary concerns, we found that a striking majority of articles are concerned with theory (n=33), followed by implementation (n=23), commentary (n=18), advocacy, ie, promoting the idea or driving the demand for an LHS (n=12), empirical data (n=11), ethics (n=8), and policy (n=7). Articles published in 2007 were exclusively theory and/or advocacy; in 2010, articles were published across a broader variety of categories. However, articles predominantly discussing ethics in our sample were only published in 2013; similarly, policy articles were only published in 2007 and 2014 (Figure 4).

Most of the implementation and empirical articles discuss the LHS in the context of oncology (n=10) [27-36]; however, other contexts include primary care (n=3) [37,38], surgery (n=3) [20,39], and pediatrics (n=7) as well as others. Some articles, such as commentary articles, exemplify clinical contexts such as pediatrics (n=3) and oncology (n=3) but are not exclusively concerned with a particular clinical context. In Table 2, we summarize the clinical contexts of articles included in our review, including the initiative of interest in each article. Articles were classified based on the objective of the literature—ie, (1) whether the article discusses or is concerned with the use of the initiative for QI, (2) whether the article discusses or is concerned with the use of the initiative for research, and/or (3) whether the article specifically evaluates the initiative with regard to improving quality or research. Most of the articles are classified as being concerned with QI, eg, around EHR point-of-care diagnostic capabilities or in clinical guidelines and subsequent process measures via networks through which information is shared. Some of these articles frame research as a stepping stone for improving point-of-care quality; however, their ultimate objective is not to improve research itself. Some studies evaluate the efficacy of QI initiatives. Fewer articles (n=9) are concerned with initiatives seeking to improve research, eg, through the development of clinical trials, or by considering the limitations in the processes through which data are collected in research used to inform practice (eg, informed consent).

**Table 2:** Clinical context of articles.

Article type, clinical context, source			Name or focus of initiative	Classification
***Implementation*** ^a^				
	**Oncology**			
		Buetow and Niederhuber [29]	Cancer Biomedical Informatics Grid	QI^b^
		Abernathy et al [28]	Rapid learning health care	QI
		Elson et al [27]	Athena Breast Health Network	QI
		Sledge et al [30]	CancerLinQ	QI
		Schilsky et al [31]	CancerLinQ	QI
		Abernathy et al [32]	Innovation in oncology	QI
	**Primary care**			
		Delaney et al [37]	Electronic Primary Care Research Network	E^c^
		Peterson et al [38]	Practice-based Research Networks	E
	**Surgery**			
		Kwon et al [39]	Surgical Care and Outcomes Assessment Program	QI/E
		Flum et al [20]	Comparative Effectiveness Research Translation Network	QI/E
	**Pediatrics**			
		Forrest et al [40]	PEDSnet consortium of 8 children’s hospitals	QI
		Forrest et al [41]	ICN^d^	QI/E
	**Endocrinology**			
		Fiore et al [42]	Point-of-care clinical trial	R^e^/E
	**Cardiology**			
		Califf and Sugarman [43]	Pragmatic clinical trials	R
		Maddox et al [44]	Veterans Administration Clinical Assessment, Reporting, and Tracking program	QI/E
	**Intensive Care Unit**			
		Warner et al [45]	Temporal phenotype data via an EHR^f^	QI/E
	**Gastro-enterology**			
		Forrest et al [41]	ICN	QI/E
	**Public Health Departments**			
		Klann et al [46]	Query Health	QI/E
	**Health systems**			
		Greene et al [47]	Rapid learning health system	QI
		Harper [48]	Clinical Demand Index	QI/E
		Weng et al [49]	Integrated Model for Patient Care and Clinical Trials	R/E
		Forrest et al [40]	PEDSnet consortium of 8 children’s hospitals	QI
		McGlynn et al [50]	Patient Outcomes Research to Advance Learning network	R
		Mandl et al [51]	Scalable Collaborative Infrastructure for a Learning Healthcare System	R/E
***Empirical***				
	**Pediatrics**			
		Lannon and Peterson [52]	Pediatric Collaborative Improvement Networks	QI/E
		Kelley et al [53]	Examining attitudes toward research	R
	**Oncology**			
		Spinks et al [33]	Not applicable	Not applicable
	**Surgery**			
		Pingleton et al [21]	Evaluating quality and patient safety curricula	QI/E
	**Nephrology**			
		Kelley et al [53]	Not applicable	Not applicable
	**Primary care and/or internal medicine**			
		Pingleton et al [21]	Evaluating quality and patient safety curricula	QI/E
***General***				
	**Pediatrics**			
		Clancy et al [22]	Collaborative Improvement Networks	QI/R
		Gardner and Kelleher [54]	LHS^g^ for Pediatrics	QI
		Kahn et al [55]	Common Pediatric Research Terminology	R/E
	**Oncology**			
		Feeley et al [34]	Health Information Technology to improve quality of cancer care	QI
		Shaikh et al [35]	Collaborative Biomedicine	QI
		Shah et al [36]	CancerLinQ	R/E

^a^Italicized categories describe concern or focus of article.

^b^QI: quality improvement.

^c^E: evaluation.

^d^ICN: ImproveCareNow.

^e^R: research.

^f^EHR: electronic health record.

^g^LHS: Learning Health System

### Themes

Our thematic analysis revealed five broad themes across the 85 articles we reviewed: (1) culture, ie, the environment or environmental change required to support the LHS; (2) innovations, including new tools or ideas needed or being developed to realize the vision of an LHS; (3) data infrastructure; and (4) ethical considerations generally framed as moral imperatives or in terms of principles such as privacy and efficiency [15]. Although some articles present evidence or discussion on multiple themes, others tend to focus exclusively on one theme, eg, articles commenting on ethical aspects. Furthermore, articles focusing on technical or research aspects rarely comment on social or ethical considerations of the LHS.

#### Culture

Most articles (n=81) set clear expectations and priorities for advancing the LHS and achieving its goals. Culture, describing the way in which research and clinical care are considered and how they contribute to the vision or mission of the LHS, is central to enhancing the system. For example, academic health centers are discussed for balancing high-quality teaching with attention to increasing customer service, productivity, and research missions and reducing knowledge gaps for evidence-based medicine [56]. In the reviewed papers, aspects of culture included (1) organizational culture; (2) research and practice; and (3) establishing a framework for health, ie, establishing the boundaries of research, practice, and QI while ensuring appropriate oversight of risks and benefits. These aspects of culture align with the initial priorities set by Etheredge in 2007, including and extending beyond leadership and collaboration, to include actionable steps such as professional education [1].

#### Innovations, Tools, and Ideas

Most of the reviewed articles (n=77) propose ideas and tools or evaluate innovations that are anticipated to contribute to achieving an LHS by (1) enhancing existing research and (2) envisioning, developing, and enhancing care methodologies to ultimately be implemented in care. For example, the Patient Outcomes Research to Advance Learning network is a research network studying the effectiveness of various approaches to diagnosis, treatment, and management, which could create cohorts of patients with common diagnoses to conduct large-scale comparative effectiveness research to accomplish its goals of assisting patients, caregivers, and physicians in making informed decisions [50]. Other tools, in contrast, are concerned with enhancing methodologies for clinical practice. For example, ImproveCareNow (ICN) is a network aiming to transform the health of children with Crohn disease and ulcerative colitis through a collaboration of pediatric gastroenterology practices working together to develop and enhance care methodologies [57]. The innovations, tools, and ideas that have been developed since 2007 seem particularly responsive to Etheredge’s envisioning of moving toward an LHS via the EHR, predictive modeling, and software development [1].

#### Data Infrastructure

Data infrastructure emerges as a key theme in articles (n=67), with authors discussing a variety of technical avenues for pursuing and/or achieving the LHS. Given that the LHS framework is heavily reliant on an emerging technical infrastructure, the significance of this theme is not surprising. Publications are concerned with (1) networks and platforms; (2) expanding study and data reliability and/or validity; and (3), particularly, the EHR as a valuable resource. In 2007, Etheredge [1] discussed the Cancer Research Network, the Vaccine Safety Datalink network, and the American Medical Group Association as exemplars of data infrastructure progressing toward an LHS. A decade later, the literature is discussing myriad networks and platforms such as the National Patient-Centered Clinical Research Network and the data-sharing platform, PopMedNet [46,50].

The literature argues that these networks and platforms rely on robust participation to build reliability and validity; however, reported participation is limited owing to inadequate sampling, limited availability of clinical information in datasets, lack of rigorous inclusion and/or exclusion criteria resulting in confounders, and resulting issues around generalizability of findings [58-60]. Furthermore, the quality of data suffers because of issues such as inconsistent terminology and the use of statistical techniques that are not advanced enough for complex observational data aggregated from the EHR [37,61]. However, articles regard the EHR as a valuable resource with the capacity to capture, communicate, aggregate, store, and analyze large pools of data for real-time clinician decision support and, broadly, for establishing a nationwide LHS [26,30,48]. Indeed, articles address limitations of the EHR spanning from the practice of individual teams building their own data repositories to the need for manual data aggregation [25,39]. However, articles urge enhancing data interoperability and tailoring the technology to specific clinical contexts such as oncology to ensure effective use of the EHR [28].

#### Ethics

According to Faden et al [23], the development of an LHS relies heavily on altruism and the understanding that although participating in research may not offer personal therapeutic or curative benefits, what is inherent to clinical research is the potential for large-scale benefits to society by filling knowledge gaps and enhancing care methods. Only eight papers focus on ethics, and most of these are part of the same special issue of the Hastings Center Report in 2013 [24,60,62]. Yet, many other papers—just under half of the reviewed papers (n=39)—discuss at least some ethical and/or social aspects of the LHS, including what is often posited as *challenges* in combining research and clinical frameworks and cultures. Specifically, articles address (1) efficacy and patient safety in research and practice, (2) the moral imperative on patients to participate in research, (3) effects of participation, eg, on privacy and the lack of guaranteed therapeutic benefits in research studies, and (4) questions of social justice (ie, fair subject selection and a just distribution of research benefits and burdens) [23,24,43,63]. For example, patients who are actively undergoing treatment may be more willing to participate in research as a result of their current experience of benefiting from treatment and scientific knowledge. However, as participants in an LHS, EHRs and other data sources will be used during, and after, specific instances of being a patient. Notions of benefit and risk are likely to change over time, both for a specific individual and within the context of a given institution. Ongoing conversations about the risks and benefits of participation, and what the idiom *research* refers to, will be critical to achieving adequate engagement and ensuring that willingness to participate is not confounded with a need for care [64,65]. Furthermore, potential users of health care services may anticipate receiving further and/or advanced treatment for long-term and rare diseases. For example, in the context of the United States, health care is not free of charge and features some of the highest costs globally for health services and long-term care. A question of social justice would be how data from patients are used for the health system to ultimately give back to patients who are charged for services and whose health and associated data may be turned into monetizable assets by health care providers.

It is a positive sign that such a significant number of articles in the sample engage with ethical and social aspects of the LHS. However, this engagement still focuses primarily on efficacy and safety and on espousing a moral duty for physicians, clinicians, and patients to participate in the LHS. In his 2007 article, Etheredge [1] discusses the ethical implications of an LHS, particularly in terms of responsibility, gaps in our understanding of comparative benefits and risks of clinical research, and prescriptions across minority and special needs groups, and raises issues of patient confidentiality. The literature, to date, concerned with ethical implications of an LHS, though sparse, continues to prioritize these gaps. Furthermore, it urges the consideration of participation and recognizes some of these issues as relating to social justice more broadly.

### Progress and Opportunities Over the Past Decade

Over the past decade, there has been tremendous movement with regard to the technical side of LHS infrastructure including the EHR, which is discussed in much of the literature as a valuable resource for research, clinical care, and the translation from one to the other [66,67]. Much of the literature in our review focuses on using EHRs for QI, signaling progress in the use and impact of health information technology on moving toward the vision of the LHS. However, our findings suggested a lack of attention to ethical considerations, eg, social justice issues, to intersections of different types of data that might enter potentially large-scale data systems such as the LHS (health, welfare, education, criminal justice, etc), and to the role, recruitment, and retention of participants in research, and equitable distribution of benefits. These aspects of health care and research have been discussed in other bodies of health literature but do not seem to have been adapted widely for and by the LHS discourse. There is also a need for further consideration of leadership, collaboration, and responsibilities of various entities within the health care system. Etheredge [1] cites Medicaid as a key leader of the LHS; for leaders to emerge, it is essential to identify, understand, and map the responsibilities of LHS leaders and identify how other stakeholders such as providers, patients, and hospitals conceptualize the LHS as a sociotechnical system.

## Discussion

### Principal Findings

Since Etheredge’s paper [1] and the US Institute of Medicine’s roundtable report in 2007 [2], more than 500 academic papers have been published discussing the LHS, with most of these emerging since 2014. This work predominantly cites empirical research describing technical developments and analytical capacity. However, LHS infrastructure concepts navigate complex systems of policy, ethics, networks, and processes in local clinical care and research settings as well as in the global consortia. Although the literature is expanding with the rate of technological innovation, an empirically based ethical analysis of large health data systems, including the deliberation of their ethical management and sociotechnical engagements, further requires considerable attention.

Articles in our review generally suggest an engagement with the LHS discourse by exploring or evaluating tools and innovations to bring the LHS framework to fruition in research and practice; by examining technical infrastructures to test the process of aggregating data from research to implement findings into practice; and by evaluating challenges and subsequent priorities to facilitate the LHS across teams and institutions. Our sample suggests that the LHS framework is considered suitable for, and is in parts already utilized across, a variety of institutions and clinical contexts, albeit not anywhere near the capacity of what the LHS framework suggests might be possible. The publication numbers themselves are indicative of the desire and effort to present, develop, and apply the LHS in action as research, technical, and clinical teams collaborate and propose new models. However, while some articles test the LHS framework as an avenue to pursuing QI, others consider the LHS a framework for implementable research. There is a need to examine whether, in applying an LHS model, a boundary exists between QI and research, and, further, the terms and implications of such a boundary. At present, the expectations and requirements for informed consent, notification, return of research results, risk assessment, privacy, and confidentiality are vastly different for research vs QI.

Future studies should examine stakeholder perceptions of the boundaries between QI and research, engaging patients, healthy participants, researchers, clinicians, and technical designers involved in LHS teams to understand the extent to which they distinguish between the two. Importantly, the ways that the LHS might be utilized need to be clarified to develop appropriate and effective governance frameworks.

Our review revealed a number of trends and themes in the LHS discourse that may inform avenues for further deliberation to contribute to a more robust discussion of the LHS. For example, the relatively small number of articles comprehensively engaging with its social, ethical, and governance aspects is surprising. This may suggest limited discussion of the LHS as a concept in the social sciences and humanities and an opportunity for further examination of aspects and implications of the LHS from these perspectives—ongoing discussions in bodies of social science scholarship around health and data would provide fruitful platforms for further discussion. The LHS relies on the collaboration of various stakeholders in research, clinical, and technical arenas to utilize technology for the improvement of patient health, in fact forming an ecosystem of interrelated and linked activities. One might expect that these interpersonal interactions as well as interactions between stakeholders and technologies would prompt further discussion of the social and ethical concerns that might arise or of the social and societal enablers, drivers, and impacts of the LHS. For example, the intersection between the patient duty to participate and the technical challenge of maintaining privacy, including data security, could be examined further to understand whether there are nuances to when patients may be more or less willing to share data across a network and to understand what the processes for decision making and data sharing need to look like to be compatible with data subjects’ needs [23].

To date, the LHS has primarily focused on the technical access between different users (via EHRs) and across local networks; this is reflected in the articles in our sample as well, suggesting a need for tests of implementation at a larger scale. ICN is an example of a regional-level network; there is the possibility of testing similar networks at a global level. It is possible that owing to our criteria of including only articles written in English, our sample excluded global-level examples of LHSs; however, it is unclear whether there are current collaborations between global settings and whether this is a part of the current LHS vision. We do know that aspects of types of LHSs—such as data governance in health care research—are being discussed in other bodies of the literature but that scholarship either does not refer to the LHS or does not take a systemic approach to health research and care.

### Implications for Future Research, Practice, and Policy

Our review suggests that although some knowledge gaps and uncertainties presented by Etheredge [1] have received consideration over the past decade, significant gaps persist, presenting opportunities for future research and policy (Table 3).

**Table 3:** Progress over the past decade in addressing knowledge gaps and uncertainties.

Knowledge gaps and uncertainties (Etheredge, 2007) [1]	Perspectives from the literature (2007-2017)	Areas for considering the way forward
Diffused responsibility	Robust discussion of the need for leadership and coordination across various stakeholders, research, policy, and practice. Tools exist for development of new technologies; however, they appear to be largely segregated into those for enhancing research vs practice, leading to disparate regulatory oversight	Further examination is needed of stakeholder perspectives of funding and responsibility for supporting research. Need for a better understanding of the roles and responsibilities of those involved in research from collection to sharing and implementation
Concerns about clinical trials	The past decade has seen a variety of innovations for enhancing research as well as clinical care, along with the emergence of networks and platforms for trials and studies across various clinical contexts. The literature sees a continued need for clinical trials to support precision medicine but asserts the need for ensuring that participants are well-informed about risks and benefits of participating, and that sampling and distribution of benefits are equitable	Continued examination of public needs, attitudes, beliefs, and knowledge about participating in research and clinical trials, especially at a systemic level of health research and care data agglomeration and learning cycles
Consequences of underinvestment	The literature suggests that the emergence of networks and platforms, as well as the increasing use of the EHR^a^ has facilitated investment in evidence-based research. However, there is a need for ensuring that data are interoperable and meaningful, that studies and data are reliable and valid, and that the public is better able to envision its role in research	Need for an enhanced design of the EHR to increase interoperability, standardization, and quality of data to ensure that findings are translatable and generalizable. Ensuring adequate regulation of research and informed consent for participants
Genetics and genomics-based medicine	Data quality, reliability, and validity are critical to genetics and genomics-based medicine. The literature primarily discusses a need for ensuring the just and equitable distribution of benefits and minimization of risks	Sampling must include patient populations beyond the socioeconomic environment of a hospital or region, and rigorous inclusion and exclusion criteria
Overall system performance	The literature extensively discusses the EHR as a resource for improving quality; several articles urge for a consideration of research vs quality improvement. In addition, there is a need for improving and developing curricula in academic medical centers on research and clinical safety and quality	Moving the LHS^b^ forward requires further theoretical consideration of research vs quality improvement. The literature is mixed and defining boundaries will be beneficial to examining the policy and practice implications of rapid learning

^a^EHR: electronic health record.

^b^LHS: Learning Health System.

Several authors have theorized and advocated the need and potential for the LHS to enhance patient safety and social justice. However, few empirical studies have actually examined the effects of immersing in an LHS culture on the patient-doctor relationship, on professional relationships in health systems, or on public accountability and governance. Generally, only a small subset of publications is acutely engaged with key ethical concerns such as trust, solidarity, equity, or privacy. Those that are engaged with these ethical concerns primarily do so from the perspective of calling attention to these concepts as potential rather than actual concerns. Given the ideal of a technical infrastructure that supports and is supported by a social and cultural learning system, it is important for studies to examine whether and how, eg, relationships between providers and administrative leaders may be different in institutions that have implemented an LHS-based technical framework. As such, we suggest that rather than considering concepts such as trust as a challenge, burden, or barrier necessary to master or overcome, the LHS discourse requires studies on how trust affects and is affected by the LHS context to understand its potential to enhance the LHS, if strengthened.

Many cited challenges and needs in our review align with governance concerns—eg, limitations of data safety and reliability, validity of data, or validity of the algorithmic outputs of data in an LHS. While this literature appears to focus on *overcoming these hurdles* through consideration of the moral obligation of researchers and participants, there was little indication of future research specifically examining the ethical and social aspects of health care and clinical research, eg, through the lens of dignity, equity, equality, and social justice. We suggest that future research needs to examine recruitment and participation in research with a particular focus on the moral imperative, to better understand implications of various approaches to enhancing participation in research on the LHS. Furthermore, the discourse around the LHS needs to attend more to the ethical implications of such systems on patients, participants, and their data and those who may share patient or participant data from both technical and ethical perspectives (eg, physicians, hospitals, public health departments, and payers). For example, it would be important to clearly understand and present practical (eg, economic and therapeutic), social, and ethical rationales for patient participation in clinical research at a systemic level of data agglomeration and analysis, outputs of which may be subject to commercial interests.

The EHR is considered a valuable resource in publications in our review, a number of articles also point specifically to this resource as requiring improvements to effectively and ethically carry through the LHS vision. Notably, EHRs are financially incentivized in the United States, making the local context ideal for implementing the LHS nationally. At the time Etheredge [1] made his call for the LHS, the Health Information Technology for Economic and Clinical Health (HITECH) Act and the US $35 billion investment in the adoption and meaningful use of EHRs had yet to materialize [68]. Since the HITECH Act, adoption and use of the EHR has continued to increase with evidence of better health outcomes after systems have had time to mature [67,69]. However, studies have also suggested consequences of EHR use on user (patient and provider) satisfaction, calling to attention the need for considering and understanding implications of the LHS on patients, and the ways in which their embeddedness in the LHS is influenced by the systemic pressures driving health information technology [70-72]. In addition, there is a need to improve ethical governance, and that continues to include necessary progress on enabling data security. Importantly, improving security does not necessarily mean restricting data from leaving the environment of a screen in an office room at a particular institution; however, there is a need to better understand the boundaries of data as understood by stakeholders including patients, clinicians, and researchers, and their rights and views on how data are retained and used. Furthermore, the information that is stored on the EHR is not always in a standardized format—eg, some data may be in the form of narrative text, whereas others may be more easily coded. For information to be usable, it is necessary to identify ways to make EHRs interoperable and, further, the information on them interpretable in a consistent and standardized way, eg, by using natural language processing techniques [73,74].

Although our review primarily focused on papers based in the United States, there is a need to examine the role and implications of an LHS in other health care system contexts. For example, the expectations and incentives for EHR use may vary across contexts. The procedures for recruitment and enrollment in research may also vary, and the principles governing use and sharing of data and health information may be different. In the US context, historical injustices in the research domain have brought to the forefront regulatory principles such as the Belmont Report describing the ethical foundations of research [75]. In addition, the influx of new actors within the LHS and big data practices expands questions related to who is doing research and innovation, including sources of funding and commercial interests. These evolutions are likely to guide the direction of work related to developing, implementing, and attending to the ethical and social considerations of an LHS [76]. As other countries and health care contexts envision and implement the LHS, it will be important to study how the histories and policies of these countries influence the LHS and how key stakeholders perceive and have expectations for what an LHS can deliver.

Finally, our sample of publications is indicative of the successes or anticipated challenges with the LHS; however, learning also results from an understanding of what goes wrong. Attempts at implementing an LHS along with evaluations of the process should be disseminated as well, for a more robust understanding of what might be optimal conditions for an LHS and what contexts may need more malleability to adopt an LHS framework and culture. For example, future studies may seek to identify the conditions under which the use of emerging technologies and delivery models achieve the goals of an LHS. For example, are the adoption of telemedicine and the use of patient-generated data via health apps and wearable devices contributing to systems that are also engaged in learning and/or feedback? Furthermore, how are the technical, social, and ethical implications of learning from clinical care delivered through telemedicine different from learning and knowledge generation that occurs through traditional, face-to-face care delivery?

### Conclusions

The past decade has observed tremendous progress in the technical infrastructure and innovative spirit of advancing the framework and implementation of the LHS. However, there are opportunities to continue to examine and develop policies ensuring the cultural and ethical imperatives for an LHS. Since Etheredge [1] presented an initial vision of the LHS in 2007, the growing implementation of the EHR and emergence of networks and platforms have contributed to the initial stages of understanding the LHS framework. However, to continue to define and realize the aims of an LHS, there is a need to consider the boundaries of research, practice, and QI. Furthermore, there is a need for engaging with various stakeholders including patients, participants, providers, and organizational leaders in clinical, technical, administrative, and research domains to understand the broader implications of an LHS.

## Abbreviations

EHR: electronic health record
HITECH: Health Information Technology for Economic and Clinical Health
ICN: ImproveCareNow
LHS: Learning Health System
QI: quality improvement
